# A Patient with Four-Year Survival after Nonsmall Cell Lung Carcinoma with a Solitary Metachronous Small Bowel Metastasis

**DOI:** 10.1155/2010/616130

**Published:** 2010-03-07

**Authors:** Klaas M. Kant, Vincent Noordhoek Hegt, Joachim G. J. V. Aerts

**Affiliations:** ^1^Department of Pulmonology, Amphia Hospital, Molengracht 21, 4300 RK Breda, The Netherlands; ^2^Department of Pathology (Pathan), Sint Franciscus Gasthuis, Rotterdam, Kleiweg 500, 3045 PM Rotterdam, The Netherlands

## Abstract

Solitary small bowel metastasis secondary to lung cancer is very uncommon. In this report, we present a patient with NSCLC and a metachronous solitary metastasis of the jejunum. She is alive without evidence of disease and doing well four years after palliative surgery, radiotherapy, and chemotherapy. To the best of our knowledge, this is the first case report describing a prolonged survival in a patient with a symptomatic solitary small bowel metastasis treated with palliative surgery, chemo- and radiotherapy instead of complete surgical resection.

## 1. Introduction

Lung cancer remains the leading cause of cancer-related death in Western countries. Nonsmall cell lung cancer (NSCLC) accounts for approximately 80% of these cases. At the time of diagnosis of NSCLC, around 40% will have stage IV disease with a median survival of 8–10 months and a 1-year survival rate of 30% [1]. Treatment with chemotherapy can be considered based on the performance status of these patients.

In the literature, unusual presentations with prolonged survival in patients with stage IV NSCLC are sporadically described, dictating a different therapeutic approach. For instance, potentially curative surgery for solitary brain metastasis or solitary adrenal gland metastasis has been described [2]. Much less is known about therapeutic choices in cases of an isolated metastasis in other organs. An example of rare metastatic site of lung cancer is the small bowel. Autopsy studies revealed small bowel metastases as the most common gastrointestinal metastasis of NSCLC, with an incidence of 4%, 6%–11% [3–6]. The number of clinical cases reporting small bowel metastasis of NSCLC is underestimated because of concurrent metastatic sites and the fact that most gastrointestinal metastases were asymptomatic (i.e., no dysphagia, abdominal pain, gastrointestinal bleeding, obstruction, intussusceptions, or perforation) [3–6]. 

In this report, we present a case of NSCLC with a metachronous solitary obstructive metastasis in the jejunum. The patient is still well and alive 4 years after subsequent treatment with palliative surgery, palliative radio- and chemotherapy. Moreover, a review of the literature on this subject is given.

## 2. Case Report

**Figure 1:**
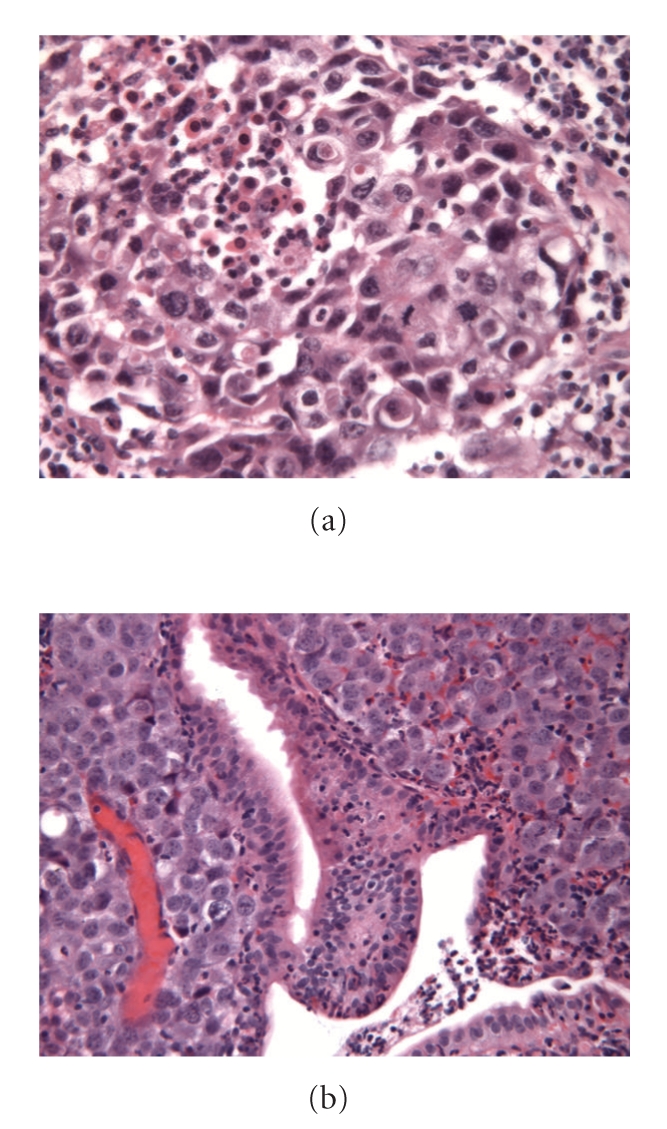
Microscopic images of the primary lung cancer and jejunal metastasis. H&E, 250X. (a), lung: a nest of large, polymorphous epithelial cells with strong mitotic activity and central necrosis is observed, consistent with large cell carcinoma (NSCLC). (b), jejunum: groups of large anaplastic cells invading the lamina propria.

A 60-year-old woman, with a history of a superficially invasive urothelial cell carcinoma of the bladder, had undergone a lung bilobectomy in January 2004 for a carcinoma in the right upper lobe. At the time of diagnosis CT of the thorax and F-18-fluoro-positron emission tomography (FDG-PET) showed no evidence of lymph node involvement or distant metastases. Mediastinal lymph nodes were not sampled during surgery. Histopathological evaluation of the resected lung tissue revealed an undifferentiated large cell carcinoma of 3,3 cm in the lung parenchyma without pleural invasion or regional lymph node metastasis, T2N0M0 (Figure 1(a)). No postoperative adjuvant treatment was recommended.

Eleven months later, the patient was referred again to our hospital because of of abdominal pain, weight loss, and fatigue. She had a performance score of 1. Physical examination showed that a mass was palpable in the left abdomen. Melena was not present at that time. All lymph node regions were found normal on palpation. Laboratory data showed no abnormalities.

**Figure 2:**
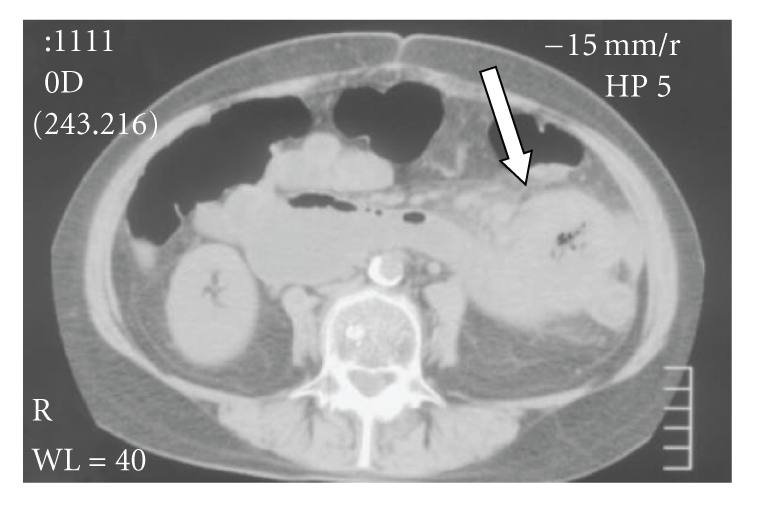
CT of the abdomen showing a solitary circular mass in the small bowel with surrounding fat infiltration.

A CT of the abdomen and double-balloon enteroscopy showed a mass in the proximal jejunum (Figure 2). Double-balloon enteroscopy, also known as push-enteroscopy, is an endoscopic technique for visualization of the small bowel. The technique involves the use of a balloon at the end of a special enteroscope camera and an overtube, which is a tube that fits over the endoscope, and which also fits with the balloon. The enteroscope and overtube are inserted as a regular gastroscope, into the small intestine. The endoscope is advanced in front of the overtube and the balloon at the end is inflated. Using the assistance of friction at the interface of the enteroscope and intestinal wall, the small bowel is accordioned back to the overtube. The overtube balloon is then deployed, and the enteroscope balloon is deflated. 

Biopsies of the jejunal mass showed groups of large undifferentiated cells in the lamina propria (Figure 1(b)). Immunohistochemical analysis revealed the tumor cells to be positive for TTF-1, cytokeratin8/ 18, cytokeratin 7, and vimentin. This immunohistochemical profile of the metastatic mass appeared comparable with the profile of previous lung tumor. It was concluded that the jejunal lesion was a metastasis of the primary lung carcinoma. This was later substantiated by mutation analysis of these two tumor tissue samples. Identical tumor-specific oncogenic K-ras gene codon 61 mutation was found in the lung tumor as well as in the jejunal metastasis. The primary urinary bladder carcinoma did not show this mutation.

**Figure 3:**
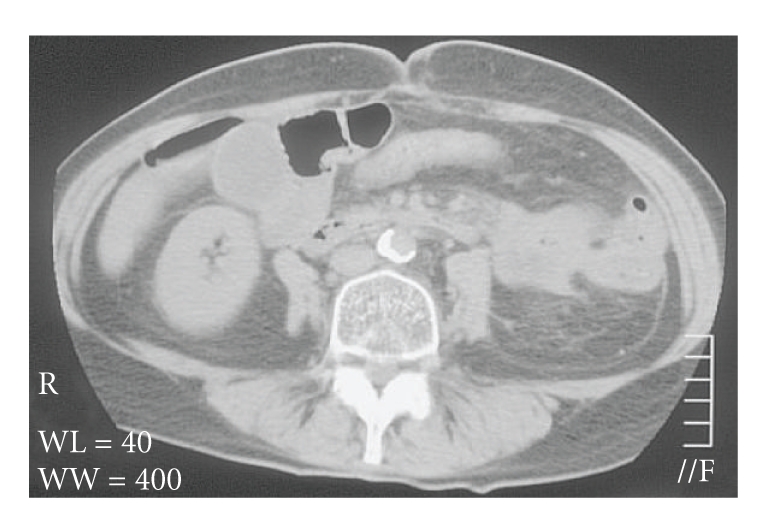
CT of the abdomen 2 months after palliative radiotherapy showing absence of residual tumor in the jejunum.

Two days after the initial histopathological diagnosis was made, intestinal obstruction occurred. Subsequent laparotomy showed a large tumor in the jejunum, which was adhered to the flexura lienalis region. Due to invasion of the mesothelium, tumor resection could not be carried out. Instead, side-to-side duodenojejunostomy was performed. Postoperatively, gastrointestinal bleeding with melena occurred. Gastroscopy showed no abnormalities. Palliative radiotherapy with a total dose of 15 GY in 5 fractions on the metastatic site was given after which the bleeding did not recur. Follow-up CT of the abdomen after 2 months showed complete absence of residual tumor in the jejunum (Figure 3).

It was chosen to further treat the patient with chemotherapy consisting of cisplatin 80 mg/m^2^ and vinorelbine 30 mg/m^2^ in a 3-week cycle. In total, she received 3 cycles. Chemotherapy was then cancelled because of serious side effects (i.e., hearing loss, presumably due to the platinum derivate).

 On follow up, the patient is doing well without any evidence of disease, 4 years after being diagnosed with a solitary metastasis of NSCLC in the jejunum.

## 3. Discussion

To the best of our knowledge, this is the first case report describing-prolonged survival in a patient with NSCLC and a symptomatic metachronous solitary small bowel metastasis treated with palliative surgery and palliative chemo- and radiotherapy instead of complete surgical resection. Rossi et al. described two patients with NSCLC and synchronous solitary small bowel metastasis who underwent small bowel resection, subsequent pulmonary lobectomy, and chemotherapy and are alive without evidence of disease [7]. 

A solitary small bowel metastasis of NSCLC is very uncommon. The jejunum seems to be the most common gastrointestinal site in these cases. The biology of a single metastatic site in patients with lung cancer remains unclear. No predominant histological cancer subtype has been characterized [4–6].

Screening for gastrointestinal metastases using CT of the abdomen has a low sensitivity. The use of whole-body FDG-PET for staging of lung cancer might increase the detection of small bowel metastases. Recently, two retrospective studies discuss the usefulness of FDG-PET in detecting gastrointestinal metastases in patients diagnosed with lung cancer [8, 9]. These studies showed that FDG-PET imaging might have a role in the detection of gastro intestinal metastases. From these limited data, no conclusions on the sensitivity of FDG-PET for establishment of small bowel metastases can be drawn. Also in the described patient retrospectively the metastatic side could not be established after reviewing the FDG-PET. 

Little can be said about survival period in patients with solitary small bowel metastasis of NSCLC as no firm data on this subject are available. Most patients described in the literature underwent surgical resection of the small bowel mass because of life-threatening complications and many times multiple metastatic sites were involved. A review by Garwood et al. describing cases with bowel perforation secondary to metastatic NSCLC showed a mean survival of 66 days [10]. Only few case reports describe small bowel localization as the unique site of metastasized NSCLC [7, 11–15]. Goh et al. reported a case series of 8 patients presenting with acute complications of gastrointestinal metastases from NSCLC [15]. Four of eight patients had a solitary metachronous metastasis small bowel. After resection of the metastasis, all four lived longer than six months. 

The choice of chemotherapy in our case remains arbitrary. Due to earlier palliative radiotherapy, residual metastatic tumor in the jejunum was not detectable anymore.Therefore, the effectiveness of chemotherapy could not be determined. It might have been solely the effect of radiotherapy on the metastatic mass that resulted in prolonged survival of our patient.

## 4. Conclusion

 A small bowel metastasis secondary to NSCLC is probably more frequent than expected, and physicians should always keep in mind this possibility. The rarity of solitary small bowel metastasis makes it difficult, if not impossible, to analyze these patient groups in randomized controlled trials. Without evidence-based medicine, an individualized approach is important.
